# Local Anaesthetic Solution

**Published:** 1913-11

**Authors:** 


Local Anaesthetic Solution. F. Chavanne recommends the following:

| | |
|---|---|
| Phenol ........................ | 2. |
| Menthol ........................ | 2. |
| Quinine hydrochlor ............ | 1.5 |
| Adrenalin ..................... | 0.005 |

This is applied on a tampon, as to the throat. The tissues shrink and become white and insensitive. Even deep cauterization can be done without pain.

